# Mechanistic Assessment of Anise Seeds and Clove Buds against the Neurotoxicity Caused by Metronidazole in Rats: Possible Role of Antioxidants, Neurotransmitters, and Cytokines

**DOI:** 10.3390/toxics11090724

**Published:** 2023-08-24

**Authors:** Amira M. El-Moslemany, Mai Hussein Abd-Elfatah, Nawal A. Tahoon, Rasha M. Bahnasy, Badriyah S. Alotaibi, Heba I. Ghamry, Mustafa Shukry

**Affiliations:** 1 Nutrition and Food Science Department, Faculty of Home Economics, Al-Azhar University, Tanta 31732, Egypt; amiraelmoslemany@azhar.edu.eg (A.M.E.-M.); rashaomar@azhar.edu.eg (R.M.B.); 2 Department of Home Economics, Faculty of Specific Education, Banha University, Banha 13511, Egypt; nawal.tahon@fsed.bu.edu.eg; 3 Department of Pharmaceutical Sciences, College of Pharmacy, Princess Nourah Bint Abdulrahman University, P.O. Box 84428, Riyadh 11671, Saudi Arabia; 4 Nutrition and Food Sciences, Department of Home Economics, Faculty of Home Economics, King Khalid University, P.O. Box 960, Abha 61421, Saudi Arabia; hgmry@kku.edu.sa; 5 Physiology Department, Faculty of Veterinary Medicine, Kafrelsheikh University, Kafrelsheikh 33516, Egypt

**Keywords:** anise seeds, clove buds, metronidazole, brain injury, antioxidant markers, neurotransmitters

## Abstract

Long-term use of the nitroimidazole-derived antibiotic metronidazole has been associated with neuronal damage due to its ability to cross the blood–brain barrier. Polyphenol-rich plants, such as anise seeds and clove buds, are suggested to have neuroprotective effects. However, their intracellular protective pathway against metronidazole-induced neurotoxicity remains unexplored. This study aims to evaluate the potential neuroprotective benefits of anise seeds and clove buds and elucidate the proposed metronidazole-induced neurotoxicity mechanism. This study divided rats into six groups, each containing six rats. In Group I, the control group, rats were administered saline orally. Group II rats received 200 mg/kg of metronidazole orally. Group III rats received 250 mg/kg b.w. of anise seed extract and metronidazole. Group IV rats received 500 mg/kg b.w. of anise seed extract (administered orally) and metronidazole. Group V rats received 250 mg/kg b.w. of clove bud extract (administered orally) and metronidazole. Group VI rats were administered 500 mg/kg b.w. of clove bud extract and metronidazole daily for 30 consecutive days. The study evaluated the phenolic compounds of anise seeds and clove buds. Moreover, it assessed the inflammatory and antioxidant indicators and neurotransmitter activity in brain tissues. A histological examination of the brain tissues was conducted to identify neuronal degeneration, brain antioxidants, and apoptotic mRNA expression. The study found that metronidazole treatment significantly altered antioxidant levels, inflammatory mediators, and structural changes in brain tissue. Metronidazole also induced apoptosis in brain tissue and escalated the levels of inflammatory cytokines. Oral administration of metronidazole resulted in a decrease in GABA, dopamine, and serotonin and an increase in ACHE in brain tissue. Conversely, oral administration of anise and clove extracts mitigated the harmful effects of metronidazole. The neurotoxic effects of metronidazole appear to stem from its ability to reduce antioxidants in brain tissue and increase nitric oxide production and apoptosis. The study concludes that neuronal damage caused by metronidazole is significantly mitigated by treatment with anise and clove extracts.

## 1. Introduction

The global use of prescribed and non-prescribed antibiotics is rising, provoking significant safety concerns. Neurological side effects associated with antibiotics are likely underdiagnosed and necessitate increased awareness and attention [1]. Metronidazole (MET) is a widely used antimicrobial drug due to its effectiveness against various bacteria and yeasts [2]. Metronidazole (MET) is generally considered a safe medication and is known to penetrate the brain rapidly. However, some potential side effects include mild abdominal pain, headaches, nausea, and a metallic taste in the mouth [3]. However, chronic exposure to metronidazole (MET) in humans at doses exceeding 2 g per day can lead to potential side effects such as peripheral neuropathy, seizures, cerebellar ataxia, and optic neuropathy [4]. Clinical symptoms associated with metronidazole-induced neurotoxicity, including ataxia, dysarthria, and altered mental status, are typical neurological manifestations [5]. For thousands of years, people across the globe have relied on traditional medicinal plants for various purposes, from maintaining overall health to treating illnesses [6]. Herbal medicine continues to be widely used as it has historically been the primary means of disease treatment. However, only a fraction of the plant kingdom has been explored for its phytochemicals. Plant extracts may contain bioactive compounds stimulating or inhibiting specific biological processes [7]. *Anise, or Pimpinella anisum* L. *(Family: Apiaceae)*, is a plant native to the Middle East and cultivated across the Mediterranean region. The fruit comprises oil, fatty acids, coumarins, flavonoids, glycosides, proteins, and carbohydrates. The seeds and essential oil have many uses, including antioxidants, antispasmodics, antimicrobials, digestive stimulants, and galactagogues. Traditional Iranian medical practitioners have even utilized aniseed to treat epilepsy and convulsions [7]. Research has demonstrated that *Pimpinella anisum* L. can mitigate lead-induced neurotoxicity [8]. *Pimpinella anisum* L. has been shown to improve memory in mice by reducing oxidative stress in the brain [9]. Clove, or Syzygium (S.) aromaticum, is a dried flower bud from the Myrtaceae family, traditionally cultivated exclusively in the Maluku Islands of Indonesia [10].

Numerous studies have demonstrated that fragrant herbs, including cinnamon, oregano, clove, thyme, and mint, possess antibacterial, antiviral, anticarcinogenic, and antifungal properties. However, clove has garnered significant attention among these spices due to its potent antimicrobial and antioxidant effects [11]. Various chemical constituents with antioxidant properties contribute to clove’s crucial role in preventing degenerative diseases [12]. Clove essential oil (CEO) can benefit multiple ailments, including burns, wounds, dental pain, tooth infections, and toothaches. It is also incorporated into soaps and perfumes and cleans histology slides. Historically, cloves have alleviated symptoms such as indigestion, abdominal pain, diarrhea, and motion sickness. In tropical Asia, cloves treat scabies, cholera, malaria, and tuberculosis. In the United States, clove has been utilized to combat food-borne infections caused by bacteria, protozoa, and even viruses and worms [12].

Furthermore, eugenol, a component of clove, has found extensive use in dentistry due to its ability to reach the bloodstream via the dental pulp tissue [13]. Sesquiterpenes, compounds isolated from clove, have been reported to possess anticarcinogenic activity [10]. In another study, Syzygium aromaticum (clove) had promising antioxidant and neuroprotective properties [14]. Due to its high concentration of bioactive compounds rich in antioxidants (phenolics, flavonoids, and tannins), the extract of Syzygium aromaticum (clove) has demonstrated significant biological benefits. It has shown potential in alleviating brain injury induced by CeCl3 and oxidative stress [15].

Clove oil (CO) is derived from the lilac plant and is primarily composed of eugenol. It has been documented that CO possesses remarkable antimicrobial and antioxidant properties [16]. Clove oil has received FDA approval and is permitted for food and medicine applications. The volatile oil extracted from clove buds exhibits significant antioxidant activity, which can be attributed to its high content of phenolic compounds, particularly eugenol and eugenol acetate, as reported by [17,18]. Clove oil can potentially be utilized in the food and pharmaceutical industries to reduce or prevent oxidation. Doing so can help hinder the formation of harmful oxidation by-products, preserve the nutritional value of products, and extend their shelf life. This application of clove oil can be beneficial in maintaining the quality and stability of both food and pharmaceutical products [19]. The antimicrobial properties of clove oil and eugenol make them suitable for inhibiting the growth of these bacteria, which are associated with oral health issues. The use of clove oil and eugenol as natural alternatives for controlling these bacterial infections (cariogenic and periodontopathogenic) could have promising implications in oral care [20]. Eugenol has the potential to restrain the assembly of essential enzymes within bacteria and cause damage to the cell wall of bacteria. These actions contribute to eugenol’s antimicrobial and hydrophobic nature and its potential as a natural antibacterial agent [21,22].

Therefore, this study aims to delineate the potential protective mechanisms of anise and/or clove essential oil that may alleviate the toxic effects induced by metronidazole. The research explores various cellular, molecular, and biochemical signaling pathways that regulate oxidative stress, apoptosis, inflammation, fibrosis, and anti-apoptotic indicators.

## 2. Materials and Methods

### 2.1. Plant Material and Animals

Anise seeds and clove buds were procured from the National Research Center located in El-Dokki, Giza, Egypt. Thirty-six male albino rats, weighing between 140–160 g, were obtained from Helwan Farm, an animal colony associated with the Vaccine and Immunity Organization in Cairo, Egypt. All animal specimens underwent thorough health checks. Before the experiment, they were allowed a week to acclimate to the laboratory environment. During this period, the rats were maintained in a calm atmosphere with natural airflow and a 12 h light–dark cycle. Food and water were provided ad libitum. The Animal Care and Use Committee of the Faculty of Veterinary Medicine at the University of Kafr El-Sheikh in Egypt approved the guidelines for the care and use of these animals.

### 2.2. Chemicals and Kits

Casein, vitamins, minerals, cellulose, choline chloride, DL-methionine, and other necessary chemicals were provided by the El-Gomhoreya Company, a provider of medications, chemicals, and medical appliances based in Cairo, Egypt. Gama Trade Company, Cairo, Egypt, supplied the kits for biochemical determinations. Both corn starch and corn oil were freshly purchased from the market in Tanta City, Al-Gharbia Governorate, Egypt. Metronidazole, under the trade name of Flagyl^®^ (250 mg tablets), was produced by Rhone-Poulenc, U.K., and supplied by the Alexandria Pharmaceutical Co., Alexandria, Egypt. The dosing and selection of metronidazole dose were based on previous research findings. According to Ogbonye et al. [23], administering metronidazole at high dosages to rats can harm the brain. It can lead to cell distortion or displacement in the cerebellum and cause damage to the cells of the pituitary gland. Metronidazole at this dose caused neurotoxicity, as shown by Oda [24].

### 2.3. Extract Preparation

The plant seeds were washed with distilled water and dried in the shade before being ground into a fine powder. This powdered substance (50 g) was added to 250 milliliters of ethanol in a beaker equipped with a magnetic stirrer, and the mixture was heated to 60 °C for 15 min. The mixture was then allowed to steep in a dark glass bottle for 24 h to ensure complete extraction. After this period, the ethanol was evaporated from the supernatant of the extracted liquid, and the resulting pure extract was filtered through a 0.2 µm membrane.

### 2.4. Phenolic Compounds Analysis

High-performance Liquid Chromatography (HPLC) was employed to separate the polyphenolic compounds of the seed extract, as per the outlined procedure. This method was used to identify the phenolic and flavonoid compounds present in the sample [25]. Once dissolved in the mobile phase, the standard phenolic acid was introduced into the HPLC system. The concentration of phenolic compounds was calculated based on the retention time and the peak area.

### 2.5. Study Model

The rats were divided into six groups, with six rats per group. In Group I, which served as the control group, the rats were administered saline orally. In Group II, designated as the metronidazole group, the rats were given orally 200 mg/kg of metronidazole dissolved in saline [23]. In Group III, the rats were given 250 mg/kg b.w. of anise seed extract and metronidazole (200 mg/kg). In Group IV, the rats were administered 500 mg/kg b.w. of anise seed extract orally, in addition to MET (200 mg/kg). In Group V, the rats were given orally 250 mg/kg b.w. of clove bud extract, along with MET. Finally, in Group VI, the rats were gavaged 500 mg/kg b.w. of clove bud extract, combined with MET, daily for 30 consecutive days. Feed intake and growth parameters were recorded every week.

### 2.6. Sampling and Biochemical Investigation

At the end of the study period, the rats were fasted overnight before being euthanized. Blood samples were collected from each rat and then centrifuged for 10 min at 3000 rpm to separate the serum. The serum was carefully transferred into dry, clean Eppendorf tubes and stored at −20 degrees Celsius for subsequent analysis, following the methodology proposed by Schermer [26]. Each rat’s brain was carefully dissected, cleaned from the adhering matter with a saline solution (0.9%), dried with filter paper, and weighed. The brain was divided into four sections: one was stored at −80 °C for later use in isolating total RNA and performing molecular analyses; another was fixed in 10% formalin for histopathological examination; a third was used fresh in the comet assay; and a fourth was homogenized for use in determining the brain’s antioxidant status.

### 2.7. Preparation of Brain Homogenates and Biochemical Analysis

The cerebrum and cerebellum were separated and washed with an ice-cold saline solution. Brain tissue was homogenized in a 1:10 (*w*/*v*) solution of ice-cold KCL buffer (1.15%, pH 7.2). The homogenate was centrifuged at 10,000× *g* for ten minutes at 4 °C, producing a post-mitochondrial supernatant (PMS). This PMS was utilized for the measurement of gamma-aminobutyric acid (GABA), following the specified methodology of Lasley et al. [27]; the level of acetylcholinesterase (AChE) was evaluated following the prescribed method by Carageorgiou et al. [28]. The levels of dopamine (DA) and serotonin (ST) were analyzed following the established protocol by Sasa and Blank [29]. The supernatant was also used to measure the concentrations of malondialdehyde (MDA), a marker for lipid peroxidation, and nitric oxide (NO), following the specified procedures by Uchiyama and Mihara [30] and Giustarini et al. [31], respectively, using ELISA plate reader at 540 nm. The enzymatic activity of Superoxide Dismutase (SOD) was determined using the method described by Marklund and Marklund [32]. Catalase (CAT) activity was measured spectrophotometrically at 240 nm by estimating the rate of H_2_O_2_ degradation, following the method described by Oberley et al. [33]. Reduced glutathione (GSH) activity was measured as previously described [34].

### 2.8. Histopathological Examination

The brain samples (cerebrum and cerebellum) were fixed in 10% formalin. They were then rinsed with tap water and placed in a dehydration bath containing successive dilutions of graded alcohol (methyl, ethyl). At 56 °C, the samples were cleaned with xylene and embedded in liquid paraffin. Using a light microscope, sections of 4 µm thickness were cut, deparaffinized, and stained with hematoxylin and eosin for histological evaluation.

### 2.9. qPCR

Gene expression in the brain was evaluated using a reverse transcription-polymerase chain reaction (RT-PCR). Approximately 100 mg of brain tissue was used to isolate total RNA using TRIzol (Invitrogen, Life Technologies, Carlsbad, CA, USA). RNA samples with a specific A260/A280 ratio greater than 1.8 were used for cDNA synthesis using a kit from Fermentas (Waltham, MA, USA). The GAPDH gene (a housekeeping gene) was amplified using the SYBR Green master mix and the primers in Table S1. The amplification data were analyzed using the 2−ΔΔT method [35].

### 2.10. Statistical Analysis

Data were analyzed using the SPSS software (SPSS Inc., Chicago, IL, USA). Statistics were reported as mean ± standard deviation. The Shapiro–Wilks test was used to test for normality. Differences between groups for normally distributed variables were analyzed using ANOVA. In cases where the analysis of variance was significant, a post hoc Tukey test was used to identify statistically substantial pairings. A *p*-value of less than 0.05 was considered statistically significant.

## 3. Results

### 3.1. The Polyphenolic Compounds of the Extract

Phenolic compounds in anise seeds and clove buds were identified using High-performance Liquid Chromatography (HPLC), with standard compounds as a reference (see Table 1). The primary components of anise, in descending order of concentration, were chlorogenic acid (153.15 µg/mL), naringenin (133.64 µg/mL), and gallic acid (63.38 µg/mL). These were followed by taxifolin, caffeic acid, ellagic acid, and syringic acid.

**Figure 1:**
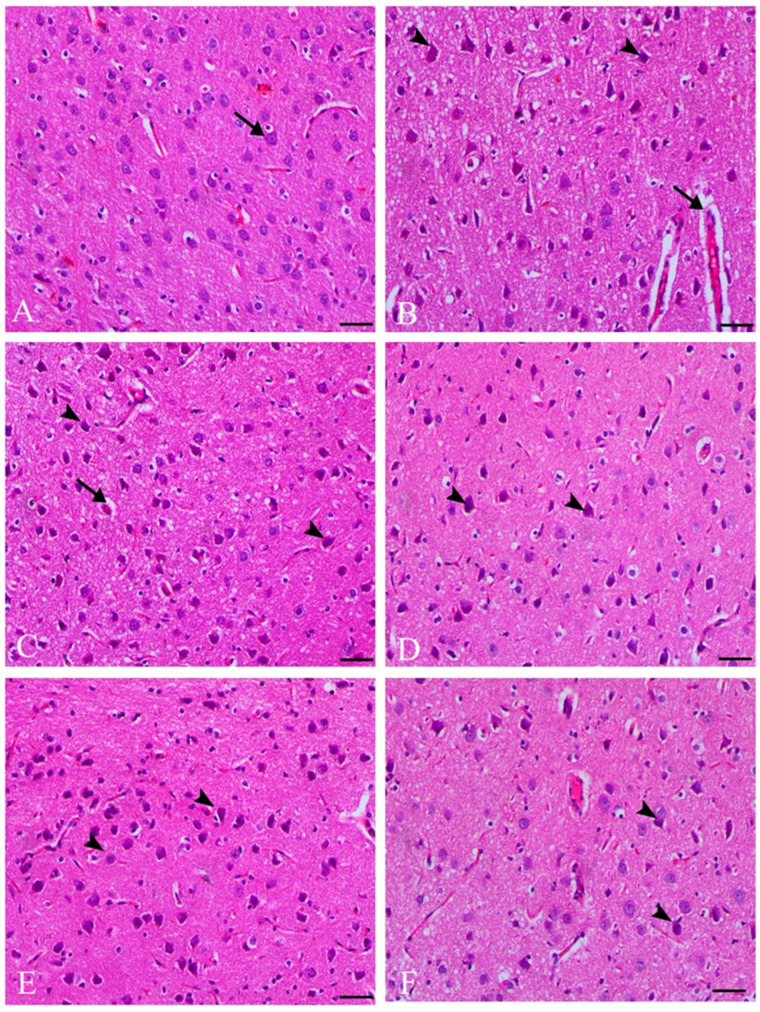
Photomicrographs of H&E stained the cerebral cortex of different treated groups—control group (**A**). The pyramidal layer of the cerebral cortex exhibited normal neuronal cells (indicated by an arrow). The metronidazole-treated group (**B**) showed severe ischemic neuronal injury in the same region, characterized by shrunken cytoplasm, nuclear pyknosis (indicated by arrowheads), and accompanied by pericellular and perivascular vacuolation (indicated by an arrow). The group treated with anise (250 mg/kg) in combination with metronidazole (**C**) showed a decrease in these ischemic neuronal degenerative changes (indicated by arrowheads) and a reduction in perivascular edema (indicated by an arrow). The group treated with anise (500 mg/kg) + metronidazole (**D**) showed decreased ischemic neuronal injury (arrowheads indicate the affected neurons). The groups treated with clove (250 mg/kg) combined with metronidazole (**E**) also displayed decreased ischemic neuronal injury (indicated by arrowheads). The group treated with clove (500 mg/kg) + metronidazole (**F**) exhibited minimal ischemic neuronal injury (indicated by arrowheads). All images were taken using H&E staining at a magnification of ×200, bar = 100 µm.

Conversely, the primary constituents of clove seeds were gallic acid (1241.87 µg/mL), catechin (257.22 µg/mL), and ellagic acid (243.16 µg/mL), followed by chlorogenic acid, syringic acid, naringenin, and pyrocatechol. Please refer to Figure 1 and Supplementary Figures S1 and S2 for the characterization of anise and clove seed extracts.

### 3.2. Body and Brain Weights

Feed intake (FI), body weight gain percentage (BWG%), and feed efficiency ratio (FER) showed a significant (*p* < 0.05) decrease in MET-treated rats compared to the control group. The administration of anise seeds and clove bud extracts in combination with MET caused a significant (*p* < 0.05) increase in these parameters compared to the MET-treated rats. Additionally, the MET-treated group displayed a notably (*p* < 0.05) lower brain weight percentage than the control group. In contrast, groups treated with seed extracts showed a significant increase in brain weight compared to the MET-treated group (see Table 2).

### 3.3. Protective Effect of Anise and Clove Seeds Extract on GABA, ACHE, DA, and ST Quantity in the Brain Rats Administered MET

Table 3 summarizes the biochemical parameters of the tested groups’ brain tissue. MET supplementation triggered a reduction of the brain GABA, DA, and ST and increased ACHE. Moreover, oral administration with anise and clove extract elevated GABA, DA, and ST suppression in brain tissue. Conversely, treated groups with anise and clove extract recorded a significant decrease in ACHE.

### 3.4. Anise and Clove Extract to Improve the Altered Antioxidant Status Due to MET

Exposure to MET significantly decreased the activity of both SOD and CAT enzymes. However, anise and clove extract showed a dose-dependent reverse effect. Anise and clove extract at two doses significantly reduced the elevated levels of MDA and NO in the MET group (*p* < 0.05). Moreover, the MET group exhibited a significant decrease in GSH levels (*p* < 0.001), which was restored by the anise and clove extract (see Table 4)

### 3.5. Histopathological Results

The metronidazole-treated group displayed severe ischemic neuronal injury in the pyramidal layer of the cerebral cortex, characterized by shrunken cytoplasm, nuclear pyknosis, and pericellular and perivascular vacuolation. However, co-treatment with anise and clove extracts and metronidazole decreased ischemic neuronal degenerative changes and reduced perivascular edema, as shown in Figure 1.

### 3.6. Effect of the Anise and Clove Extract on the Antioxidants and Apoptotic Gene Expression of the Brain Subjected to MET

**Figure 2:**
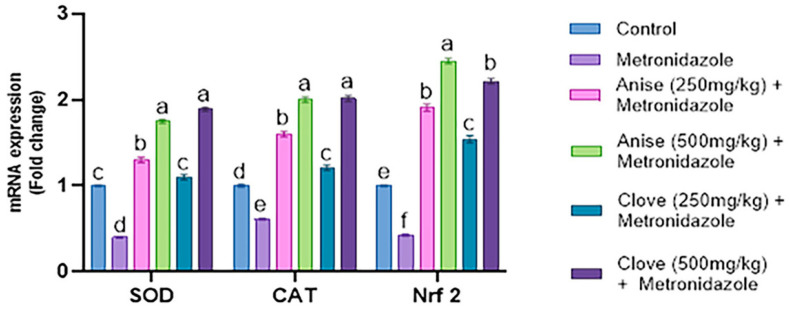
Effect of anise and clove seeds extract on the SOD, CAT, and Nrf2 mRNA expression in MET-treated male rats. Data are shown as mean ± SEM. The data were analyzed using one-way ANOVA, followed by Bonferroni multiple comparisons test. ^a,b,c,d,e,f^ Values with different letters are statistically different at *p* < 0.05. (N = 5).

Our results demonstrated that oral administration of MET significantly downregulated the mRNA expression of SOD, CAT, and Nrf2 compared to the other treated groups. In contrast, the groups treated with anise and clove extract showed a significant upregulation of SOD, CAT, and Nrf2 mRNA expression compared to the MET-treated group. Notably, the larger doses of anise and clove extract led to a pronounced increase in the expression of these genes. These findings are illustrated in Figure 2.

**Figure 3:**
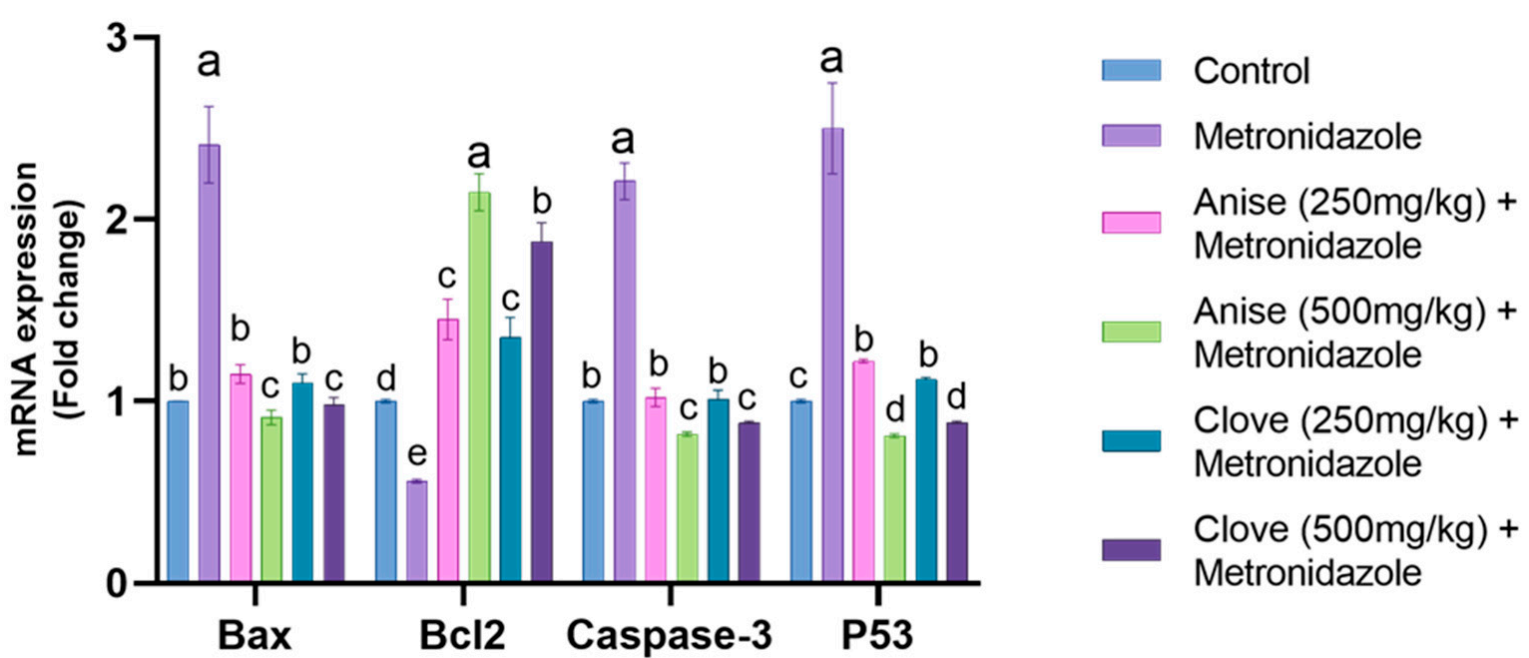
Effect of anise and clove seeds extract on the Bax, Bcl2, Caspase-3, and P53 mRNA expression in MET-treated male rats. Data are shown as mean ± SEM. The data were analyzed using one-way ANOVA, followed by Bonferroni multiple comparisons test. ^a,b,c,d,e^ Values with different letters are statistically different at *p* < 0.05. (N = 5).

As depicted in Figure 3, a significant upregulation in the mRNA expression of Bax, caspase 3, and P53 was observed, coupled with a substantial decrease in the mRNA expression of Bcl2 compared to the other treated groups. In contrast, the groups treated with anise and clove extract showed a notable downregulation of the mRNA expression of Bax, caspase-3, and P53 compared to the MET-treated group. This was accompanied by a pronounced increase in the mRNA expression of Bcl2, with the larger doses of anise and clove extract showing a marked improvement.

## 4. Discussion

Long-term use of metronidazole, a nitroimidazole-derived antibiotic, has been associated with neuronal damage due to its ability to cross the blood–brain barrier. However, chronic use of metronidazole in humans at doses at or above 2 g per day can result in potential side effects, including peripheral neuropathy, seizures, cerebellar ataxia, and optic neuropathy [4]. Metronidazole can cause a range of neurological syndromes, including cerebellar syndrome, encephalopathy, seizures, optic neuropathy, autonomic neuropathy, and peripheral neuropathy [36]. Adverse effects on the central nervous system due to metronidazole toxicity have been observed in humans and various animal species, such as dogs, rats, and cats. Additionally, there have been reports of cerebellar syndrome occurring due to prolonged exposure to metronidazole [24]. A previous study has provided clear evidence that metronidazole can cross the blood–brain barrier and accumulate in specific brain regions, including the hippocampus, olfactory bulb, and cerebellum [37]. Metronidazole can lead to adverse effects on the central nervous system, resulting in a condition known as metronidazole-induced encephalopathy [5]. Later, Evans et al. [38] GABA (gamma-aminobutyric acid) has been identified as the main inhibitory neurotransmitter in the cerebellar and vestibular systems, which are affected by metronidazole intoxication.

Herbal treatments are gaining increasing recognition due to their natural origins, perceived safety, and demonstrated effectiveness against a broad spectrum of health conditions, including cardiovascular disease, diabetes, and cancer [39,40]. The seeds of *Pimpinella anisum* L., commonly known as anise, have been extensively researched for their numerous beneficial effects on human health [41]. Their medicinal properties include functioning as a diuretic, an antihypertensive, an anti-diabetic, an anti-cancer, an immunomodulator, an antibiotic, an anti-inflammatory, an analgesic, and even an anti-stress agent [42]. The natural phenolic antioxidant properties found in clove bud extract make it an effective treatment for disorders caused by oxidative stress [43]. Various compounds extracted from alcoholic and aqueous extracts of clove buds, such as tannins, ellagic acid, gallic acid, flavonoids, and their glycosides, have been attributed with numerous health benefits. These include antithrombotic, antiprotozoal, hypoglycemic, anti-inflammatory, gastro-protective, and aphrodisiac effects [44]. Anise seeds and clove bud extract, which contain highly bioactive phenolic components, have been identified as potential treatments for the neurotoxic effects of MET.

In the MET-treated group, adverse effects on brain function parameters (GABA, DA, and ST) were observed, along with increases in ACHE and the brain level of oxidative stress markers (MDA, NO). There were also decreases in the activity of brain antioxidant enzymes (SOD, CAT, and GSH). These findings agree with Tahoun [45], who demonstrated that significant weight loss and the onset of neurological symptoms were induced by daily doses of 500 mg/kg body weight of metronidazole for 60 days. These results do not align with the findings of Sohrabi [46], who demonstrated that a dose of 400 mg/kg of metronidazole had no significant impact on body weight after 60 days. Additionally, Chukwu et al. [47] showed that administering metronidazole at 200 and 400 mg/kg doses for 28 days did not affect body weight. These discrepancies could explain variations in metronidazole dosage and study duration. It could also be attributed to anorexia (lack of appetite) in the animals, as was observed and corroborated in an earlier investigation [48]. While several theories have been proposed to explain how metronidazole (MTZ) causes cerebellar toxicity, the exact mechanism remains unclear. One hypothesis suggests that MTZ and its metabolites cause reversible axonal swelling and symmetrical damage to the cerebellar nuclei by binding to neuronal RNA and inhibiting protein synthesis [49]. Another theory posits that the inhibitory neurotransmitter gamma-aminobutyric acid (GABA) receptors in the vestibular and cerebellar systems may also be influenced by MTZ [38]. In addition, MTZ may stimulate the production of harmful radicals, including semiquinone and nitro anion radicals [50].

Congestion and some displacement of Purkinje cells were also noted in experimental mice treated with 200 mg/kg/day of metronidazole (MTZ). This observation suggests that chronic exposure to escalating doses of metronidazole may result in cerebellar damage, as highlighted in a study by Agarwal et al. [51]. Metronidazole has been demonstrated to have toxicological effects on brain cells [52]. Biochemical evaluations in brain tissue indicated elevated levels of MDA and NO in the metronidazole (MET)-treated group compared to the control group, with the increase being statistically significant (*p* < 0.05). Researchers discovered that mice administered with metronidazole experienced increased MDA activity, leading to higher lipid peroxidation (LPO) levels, a marker suggestive of potential cellular damage [53].

Metronidazole significantly affects the activity of brain antioxidant enzymes such as SOD, CAT, and GSH. Additionally, it dramatically alters inflammatory mediators and induces morphological changes [3]. The gene expression data from our study supports these findings, demonstrating that oral administration of MET significantly suppressed the mRNA expression of SOD, CAT, and Nrf2 compared to other treatment groups. Neurons in the MET group exhibited severe ischemic injury, characterized by reduced cytoplasm, nuclear pyknosis, and pericellular and perivascular vacuolation. In addition, Purkinje cells selectively underwent cell death and showed signs of degeneration, including swelling, vacuolation, and clumping of protoplasm [24].

Additionally, the results indicated that treatment with anise seeds and clove buds extract improved tissue levels of brain function parameters (GABA, DA, and ST), decreased ACHE, and increased feed intake, body weight gain percentage, feed efficiency ratio, and brain weight percentage. Improvements in brain histology and a reduction in the concentration of oxidative stress markers (MDA and NO) were also observed. These results aligned with Cabuk et al. [54], which demonstrated a substantial increase in body weight following treatment with 750 mg/kg of *Pimpinella anisum* L. aqueous extract for 15 days.

The bio-active compounds in aniseed—including Anethole, Eugenol, Anisaldehyde, Estragol, and Methylchavicol—may stimulate the digestive system and contribute to this effect. Anethole, the primary compound in *Pimpinella anisum* L., has been found to inhibit the growth of pathogenic microorganisms in the digestive tract, leading to improved weight gain and feed conversion. Anise seeds contain a volatile oil that comprises 1.5–6% of their makeup, with trans-anethole making up 88% of this oil [55]. Cabuk et al. [56] suggested that the oil extracted from anise seeds could potentially modify the performance of medications targeting the central nervous system. According to our results, anise seeds effectively reduce lipid peroxidation and enhance the activity of antioxidant enzymes (SOD, CAT, and GSH). The radical scavenging phytochemicals present in these plants may be responsible for this effect. Therefore, Bekara et al. [7] demonstrated the effectiveness of polyphenols in reducing lipid peroxidation. Polyphenols are a class of bioactive compounds found in nearly all plant species. Multiple studies have shown that the polyphenols in *Pimpinella anisum* L. seeds donate an electron to free radicals and then react with them to form more stable compounds. This process restores the pro-oxidant/antioxidant balance, thereby reducing lipid peroxidation [57].

Anise and clove extract caused a considerable upregulation of SOD, CAT, and Nrf2 mRNA expression compared to the MET-treated group. A pronounced enhancement in the expression of these genes was observed at higher dosages of the anise and clove extract, corroborating our results. In addition, the essential oil of this plant may function as an effective antidepressant [58]. *Pimpinella anisum* L. is preventive against the development of cerebrovascular diseases and is therapeutic in treating neurological conditions such as epilepsy and seizures [59]. Seeds that contain anise oil have demonstrated a neuroprotective effect, likely through enhanced modulation of NMDA activities, including activating the glycine site NMDA receptor [60]. Anise oil, possibly by activating GABA A receptors, exhibits an inverse effect, reducing hyper-locomotor activity [58]. Treatment with anise seed extract alleviated the histological deterioration induced by MET. The overall cellular structure improved following the anise seed extract treatment, although vacuolization persisted [7]. In addition, [61] demonstrated the inhibition of AChE by clove extract.

Furthermore, eugenol and isoeugenol mitigated the increase in AChE activity and intracellular Ca^2+^ levels in the cerebral cortex and cerebellum regions of rat brains after exposure to acrylamide [62]. However, treatment with clove bud extract counteracted the toxicity of MET by enhancing the activity of CAT, SOD, and GSH. The results align with Gülçin et al. [63], who discovered that clove buds are rich in antioxidants. Concurrently, the physiological functions of CAT, SOD, and GPx are interconnected. SOD catalyzes the conversion of superoxide anions (O_2_) into molecular oxygen and hydrogen peroxide. Subsequently, catalase and peroxidase cooperate to convert H_2_O_2_ into water. Reduced activity of SOD is consistent with lower levels of intracellular H_2_O_2_, which consequently reduces CAT and GPx activity [64]. The antioxidant status may have been restored due to the ability of clove components to scavenge free radicals induced by artesunate stress. When S. aromaticum bud extract was administered, the antioxidant status improved and lipid levels decreased [65]. Amber et al. [14] demonstrated the antioxidant and neuroprotective potential of S. aromaticum. AlCl3-treated rats on a diet including clove aqueous extract showed a significant increase in their GSH content and GPx activity after 14 days of daily gavage [62]. In addition, eugenol, a component of clove, exhibits anti-oxidative and anti-amyloid beta peptide activity, as well as cholinomimetic action. Studies on mice have shown that acute treatment with an ethanolic clove extract enhances learning and memory recall [66].

The mRNA levels of Bax, caspase-3, and P53 were significantly upregulated, while the expression of Bcl2 mRNA was notably downregulated. These findings are consistent with previous studies on MET-treated rats [3]. Furthermore, in the anise-treated group, the high estrogen level—known for its anti-apoptotic action—was restored, along with the downregulation of caspase 3. This provides further evidence that MET induces apoptosis in neuronal cells. Given that several components in aniseed possess antioxidant properties, it may be reasonable to consider it a significant natural estrogen source [67]. In addition, clove extract mitigates oxidative stress and cellular apoptotic death [68,69].

## 5. Conclusions

This study illustrates how MET is associated with modifications in various biomarkers, including oxidative stress markers, inflammatory mediators, determinants of apoptosis, neurotransmitters, and nitric oxide signaling molecules. The present research reveals the neuroprotective qualities of anise seeds and clove buds when co-administered with MET at diverse doses. While further exploration is needed to elucidate the precise mechanism underpinning MET-induced neuronal degeneration, this work may lay the groundwork for future investigations.

## Figures and Tables

**Table 1:** Phenolic compounds of anise and clove seed extract (µg/mL).

	Anise (1 g/15 mL)		Clove (1 g/15 mL)	
Compounds				
	Area	Conc. (µg/mL)	Area	Conc. (µg/mL)
Gallic acid	721.26	63.38	14,131.40	1241.87
Chlorogenic acid	2050.14	153.15	3252.87	243.00
Catechin	28.44	3.41	2029.36	243.16
Methyl gallate	0.00	0.00	1014.85	13.78
Coffeic acid	1085.13	46.38	0.00	0.00
Syringic acid	475.21	20.80	4503.74	197.08
Pyro catechol	202.00	14.33	473.29	33.58
Rutin	89.69	12.21	10.48	1.43
Ellagic acid	645.97	43.22	3844.39	257.22
Coumaric acid	48.63	0.83	1967.15	33.44
Vanillin	259.17	5.83	103.12	2.32
Ferulic acid	66.09	2.31	0.00	0.00
Naringenin	2417.75	133.64	2310.65	127.72
Taxifolin	800.27	58.17	313.15	22.76
Cinnamic acid	570.17	5.76	220.99	2.23
Kaempferol	9.14	0.36	20.42	0.80

**Table 2:** Protective effect of anise and clove seeds extract on FI (g/d), BWG%, FER, and brain weight % in MET-treated male rats.

Groups	FI (g/d)	BWG %	FER	Brain %
Control	23.35 ± 0.28 ^a^	29.90 ± 2.12 ^a^	0.19 ± 0.01 ^a^	1.58 ± 0.13 ^a^
Metronidazole	17.87 ± 0.12 ^e^	8.85 ± 0.23 ^e^	0.02 ± 0.005 ^d^	0.76 ± 0.04 ^c^
Anise (250 mg/kg) + Metronidazole	22.49 ± 0.12 ^ab^	26.17 ± 0.64 ^b^	0.04 ± 0.002 ^c^	0.97 ± 0.01 ^b^
Anise (500 mg/kg) + Metronidazole	20.95 ± 0.64 ^c^	23.94 ± 0.68 ^c^	0.05 ± 0.004 ^c^	1.56 ± 0.98 ^a^
Clove (250 mg/kg) + Metronidazole	19.87 ± 0.94 ^d^	22.47 ± 1.02 ^c^	0.07 ± 0.003 ^b^	1.02 ± 0.003 ^b^
Clove (500 mg/kg) + Metronidazole	21.79 ± 0.19 ^bc^	19.49 ± 1.55 ^d^	0.06 ± 0.005 ^b^	0.95 ± 0.133 ^b^

The data are presented as mean ± SD. The statistical analysis was performed using one-way ANOVA followed by the Tukey multiple range test. ^a,b,c,d,e^ means within the same column having different superscripts indicate statistically significant differences (*p* < 0.05).

**Table 3:** Protective effect of anise and clove seeds extract on GABA, ACHE, DA, and ST levels in MET-treated male rats.

Groups	GABA (Pg/mL)	ACHE (Pg/mL)	DA (ng/mL)	ST (ng/mL)
control	404.02 ± 22 ^a^	11.21 ± 21 ^e^	2.51 ± 0.12 ^a^	67.5 ± 6.5 ^a^
Metronidazole	46.86 ± 4.26 ^f^	87.12 ± 6.1 ^a^	0.15 ± 0.005 ^f^	5.21 ± 0.9 ^e^
Anise (250 mg/kg) + Metronidazole	97.41 ± 12.9 ^e^	67.35 ± 2 ^b^	0.37 ± 0.01 ^e^	25.31 ± 6.8 ^d^
Anise (500 mg/kg) + Metronidazole	274.75 ± 26.55 ^c^	20.51 ± 3.5 ^d^	0.94 ± 0.03 ^c^	50.41 ± 20 ^c^
Clove (250 mg/kg) + Metronidazole	157.5 2 ± 9.5 ^d^	42.51 ± 5.5 ^c^	0.76 ± 0.04 ^d^	32.21 ± 5.7 ^d^
Clove (500 mg/kg) + Metronidazole	335.51 ± 10.5 ^b^	19.11 ± 1.1 ^d^	1.12 ± 0.03 ^b^	59.14 ± 1.2 ^b^

The data are presented as mean ± SD. The statistical analysis was performed using one-way ANOVA followed by the Tukey multiple range test. ^a,b,c,d,e,f^ means within the same column having different superscripts indicate statistically significant differences (*p* < 0.05).

**Table 4:** Effect of anise and clove seeds extract on MDA, NO, CAT, SOD, and GSH levels in MET-treated male rats.

Groups	MDA (nmol/gm)	NO (umol/L)	SOD (U/mg)	CAT (U/mg)	GSH (mg/gm)
Control	1.24 ± 0.51 ^f^	5 ± 1 ^e^	222 ± 16 ^a^	9.91 ± 1.09 ^a^	192.5 ± 11.5 ^a^
Metronidazole	25.75 ± 2 ^a^	86 ± 7 ^a^	30.5 ± 2.5 ^e^	0.59 ± 0.03 ^d^	24.5 ± 2.5 ^d^
Anise (250 mg/kg) + Metronidazole	18.6 ± 1.12 ^b^	45 ± 5 ^c^	48.5 ± 9.5 ^d^	2.22 ± 0.26 ^c^	70 ± 3 ^c^
Anise (500 mg/kg) + Metronidazole	9.92 ± 0.92 ^d^	19.5 ± 1.5 ^d^	130 ± 9 ^b^	5.52 ± 0.72 ^b^	95 ± 5 ^b^
Clove (250 mg/kg) + Metronidazole	12.65 ± 0.15 ^c^	56.5 ± 6.5 ^b^	80.5 ± 0.13 ^c^	1.86 ± 0.5 ^c^	73.5 ± 5.74 ^c^
Clove (500 mg/kg) + Metronidazole	6.45 ± 1.39 ^e^	27.5 ± 3.5 ^d^	126 ± 7 ^b^	5.08 ± 0.82 ^b^	93 ± 3 ^b^

The data are presented as mean ± SD. The statistical analysis was performed using one-way ANOVA followed by the Tukey multiple range test. ^a,b,c,d,e,f^ means within the same column having different superscripts indicate statistically significant differences (*p* < 0.05).

## Data Availability

Not applicable.

## Supplementary Materials

The following supporting information can be downloaded at: https://www.mdpi.com/article/10.3390/toxics11090724/s1, Figure S1: HPLC of Anise characterization; Figure S2: HPLC of Clove characterization; Table S1: Primers for gene expression by RT-PCR.
