# Supplementary material for: Mechanistic Assessment of Anise Seeds and Clove Buds against the Neurotoxicity Caused by Metronidazole in Rats: Possible Role of Antioxidants, Neurotransmitters, and Cytokines
Source: Toxics. 2023 Aug 24;11(9):724. doi: 10.3390/toxics11090724 (PMC10538152; doi:10.3390/toxics11090724)
Supplement: Supplementary file 1 [file toxics-11-00724-s001.zip › toxics-2541621-supplementary.pdf]

Data File C:\CHEM32\1\DATA\POLYPHENOLS 10-1-2021 2021-01-10 13-10-37\013-1601.D
Sample Name: Anise characterization **(Figure S1)**

===
Acq. Operator : SYSTEM Seq. Line : 16
Acq. Instrument : hplc -2 Location : Vial 13
Injection Date : 10/01/2021 06:49:56 م Inj : 1
Inj Volume : 10.000 µl
Acq. Method : C:\CHEM32\1\DATA\POLYPHENOLS 10-1-2021 2021-01-10 13-10-37\POLYPHENOL 2017.M
Last changed : 10/01/2021 03:38:23 م by SYSTEM
Analysis Method : C:\CHEM32\1\DATA\POLYPHENOLS 10-1-2021 2021-01-10 13-10-37\POLYPHENOL 2017.M (Sequence Method)
Last changed : 12/01/2021 03:33:38 م by SYSTEM
(modified after loading)
Additional Info : Peak(s) manually integrated

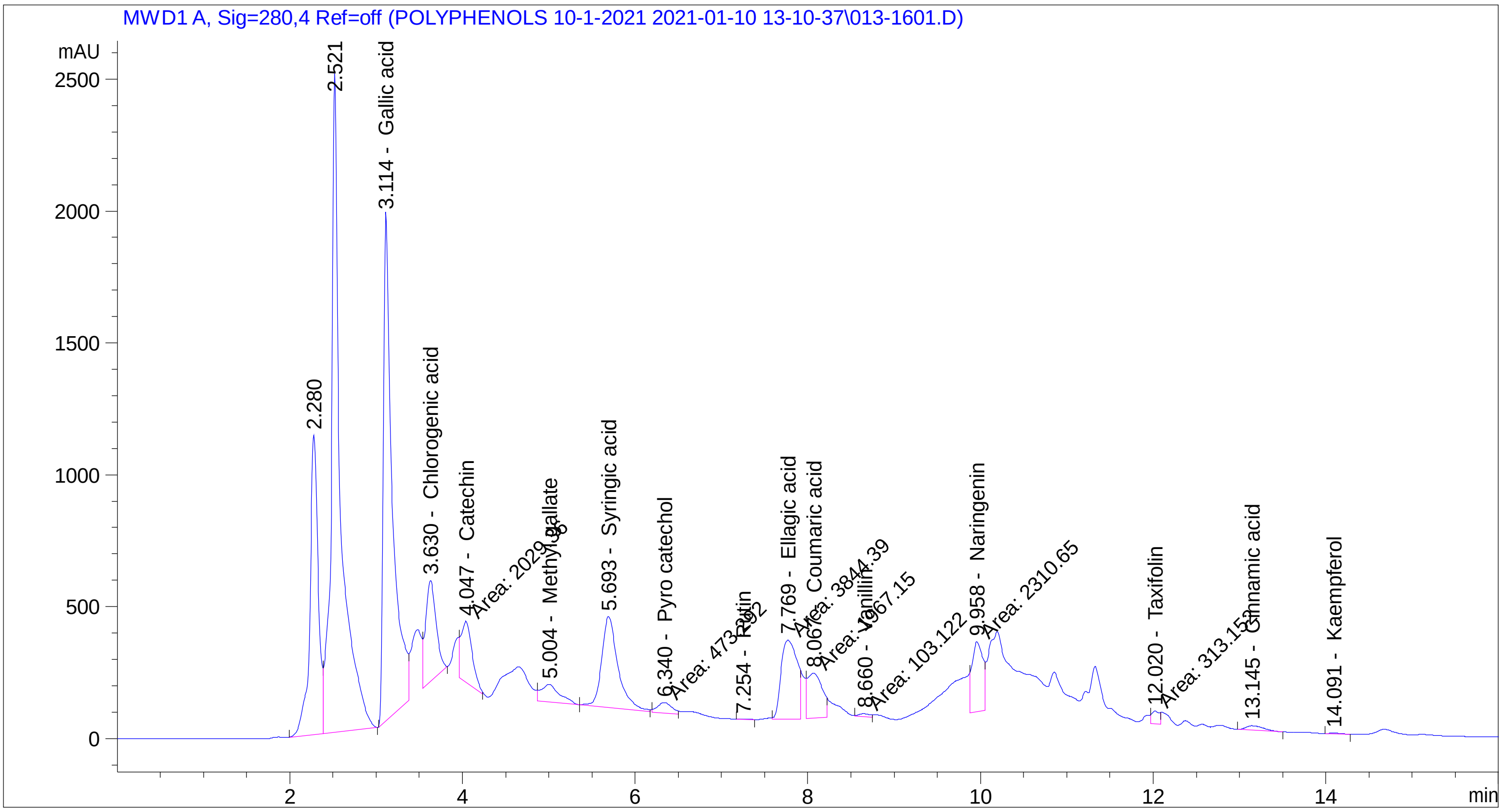

===
Area Percent Report
===

Sorted By : Signal
Calib. Data Modified : 12 January, 2021 03:33:38 م
Multiplier : 1.0000
Dilution : 1.0000
Use Multiplier & Dilution Factor with ISTDs

Signal 1: MWD1 A, Sig=280,4 Ref=off

Peak #	RetTime [min]	Type	Width [min]	Area [mAU*s]	Area %	Name
1	2.280	BV	0.1006	7638.87354	12.5413	?
2	2.521	VB	0.1031	1.90750e4	31.3168	?
3	3.114	BV	0.1035	1.41314e4	23.2005	Gallic acid
4	3.630	VB	0.1270	3252.87256	5.3405	Chlorogenic acid

Peak #	RetTime [min]	Type	Width [min]	Area [mAU*s]	Area %	Name
5	4.047	MM	0.1464	2029.35522	3.3317	Catechin
6	5.004	VB	0.2065	1014.84912	1.6662	Methyl gallate
7	5.428		0.0000	0.00000	0.0000	Coffeic acid
8	5.693	BV	0.1923	4503.74121	7.3941	Syringic acid
9	6.340	MM	0.1970	473.29214	0.7770	Pyro catechol
10	7.254	BB	0.1250	10.47772	0.0172	Rutin
11	7.769	MM	0.2140	3844.38647	6.3116	Ellagic acid
12	8.067	MM	0.1928	1967.14709	3.2296	Coumaric acid
13	8.660	MM	0.1485	103.12163	0.1693	Vanillin
14	9.485		0.0000	0.00000	0.0000	Ferulic acid
15	9.958	MM	0.1450	2310.64795	3.7936	Naringenin
16	12.020	MM	0.1094	313.15201	0.5141	Taxifolin
17	13.145	BB	0.2206	220.99344	0.3628	Cinnamic acid
18	14.091	BB	0.1271	20.41864	0.0335	Kaempferol

Totals : 6.09097e4

2 Warnings or Errors :

Warning : Calibration warnings (see calibration table listing)
Warning : Calibrated compound(s) not found

===

*** End of Report ***

Data File C:\CHEM32\1\DATA\POLYPHENOLS 10-1-2021 2021-01-10 13-10-37\014-1701.D
Sample Name: **Clove characterization Figure S2**

===

Acq. Operator : SYSTEM Seq. Line : 17
Acq. Instrument : hplc -2 Location : Vial 14
Injection Date : 10/01/2021 07:12:27 م Inj : 1
Inj Volume : 10.000 µl
Acq. Method : C:\CHEM32\1\DATA\POLYPHENOLS 10-1-2021 2021-01-10 13-10-37\POLYPHENOL 2017.M
Last changed : 10/01/2021 03:38:23 م by SYSTEM
Analysis Method : C:\CHEM32\1\DATA\POLYPHENOLS 10-1-2021 2021-01-10 13-10-37\POLYPHENOL 2017.M (Sequence Method)
Last changed : 12/01/2021 03:33:38 م by SYSTEM
(modified after loading)
Additional Info : Peak(s) manually integrated

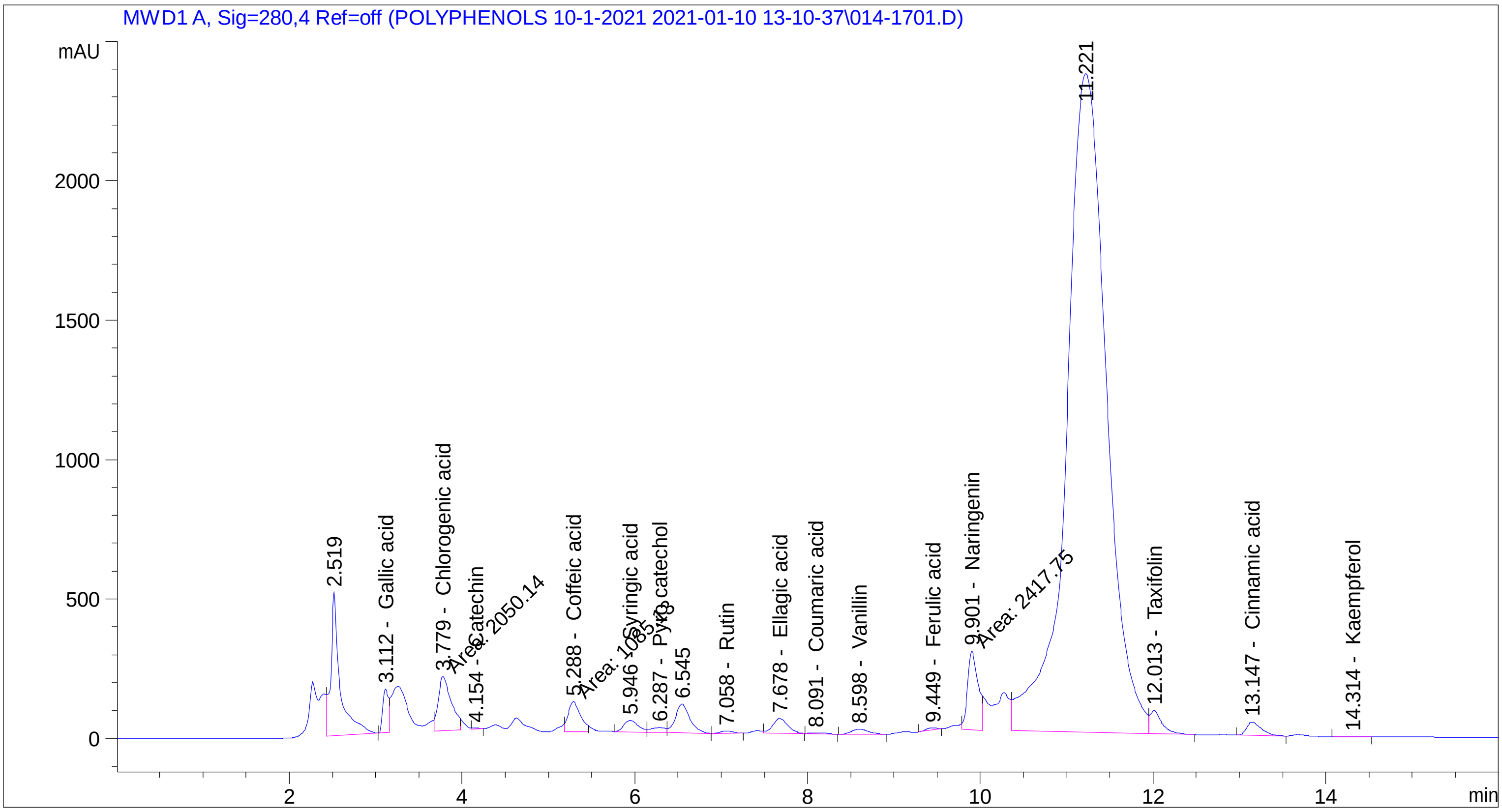

===

Area Percent Report

===

Sorted By : Signal
Calib. Data Modified : 12 January, 2021 03:33:38 م
Multiplier : 1.0000
Dilution : 1.0000
Use Multiplier & Dilution Factor with ISTDs

Signal 1: MWD1 A, Sig=280,4 Ref=off

Peak #	RetTime [min]	Type	Width [min]	Area [mAU*s]	Area %	Name
1	2.519	VB	0.0993	3833.98291	4.2273	?
2	3.112	BV	0.0696	721.25635	0.7953	Gallic acid
3	3.779	MM	0.1750	2050.14233	2.2605	Chlorogenic acid
4	4.154	VB	0.0834	28.44088	0.0314	Catechin

Peak #	RetTime [min]	Type	Width [min]	Area [mAU*s]	Area %	Name
5	5.037		0.0000	0.00000	0.0000	Methyl gallate
6	5.288	MM	0.1670	1085.13452	1.1965	Coffeic acid
7	5.946	BV	0.1788	475.21027	0.5240	Syringic acid
8	6.287	VV	0.1740	202.00060	0.2227	Pyro catechol
9	6.545	VB	0.1744	1213.27148	1.3377	?
10	7.058	BB	0.1623	89.68539	0.0989	Rutin
11	7.678	VB	0.1843	645.96545	0.7122	Ellagic acid
12	8.091	BB	0.2345	48.63359	0.0536	Coumaric acid
13	8.598	BB	0.2069	259.16736	0.2858	Vanillin
14	9.449	BB	0.1481	66.08925	0.0729	Ferulic acid
15	9.901	MM	0.1427	2417.74707	2.6658	Naringenin
16	11.221	VV	0.4965	7.61792e4	83.9945	?
17	12.013	VB	0.1355	800.26587	0.8824	Taxifolin
18	13.147	VV	0.1756	570.16901	0.6287	Cinnamic acid
19	14.314	BB	0.2090	9.14474	0.0101	Kaempferol

Totals : 9.06955e4

2 Warnings or Errors :

Warning : Calibration warnings (see calibration table listing)
Warning : Calibrated compound(s) not found

===

*** End of Report ***

Table S1. Primers for gene expression by RT-PCR

Gene	Direction	Primer sequence	Accession number
CAT	Sense	TAAGACTGACCAGGGCA	NM_012520.2
	Antisense	CAAACCTTGGTGAGATCGAA	
CASP3	Sense	CTGGACTGCGGTATTGAGAC	NM_012922.2
	Antisense	CCGGGTGCGGTAGAGTAAGC	
Bax	Sense	GGCGAATTGGCGATGAACTG	NM_017059.2
	Antisense	ATGGTTCTGATCAGCTCGGG	
Bcl-2	Sense	GATTGTGGCCTTCTTTGAGT	NM_016993.1
	Antisense	ATAGTTCCACAAAGGCATCC	
SOD	Sense	AGGATTAACTGAAGGCGAGCAT	NM_017050.1
	Antisense	TCTACAGTTAGCAGGCCAGCAG	
Nrf2	Sense	TGTCAGCTACTCCCAGGTTG	NM_031789.2
	Antisense	ATCAGGGGTGGTGAAGACTG	
GAPDH	Sense	TCAAGAAGGTGGTGAAGCAG	NM_017008.4
	Antisense	AGGTGGAAGAATGGGAGTTG	

Bax, Bcl-2-associated X protein. Bcl-2, B-cell lymphoma 2. CASP3, caspase 3. CAT, catalase. GAPDH, glyceraldehyde-3-phosphate dehydrogenase. SOD, superoxide dismutase. (Nrf2) Nuclear factor (erythroid-derived 2)-like 2.
